# Supplementary material for: Spatial integration of sensory input and motor output in Pseudomonas aeruginosa chemotaxis through colocalized distribution
Source: eLife. 2025 Sep 4;13:RP97514. doi: 10.7554/eLife.97514 (PMC12410967; doi:10.7554/eLife.97514)
Supplement: Figure 3—source data 1. [file elife-97514-fig3-data1.zip › Figure 3B-Source data 1/Figure 3B-source data 1.pdf]

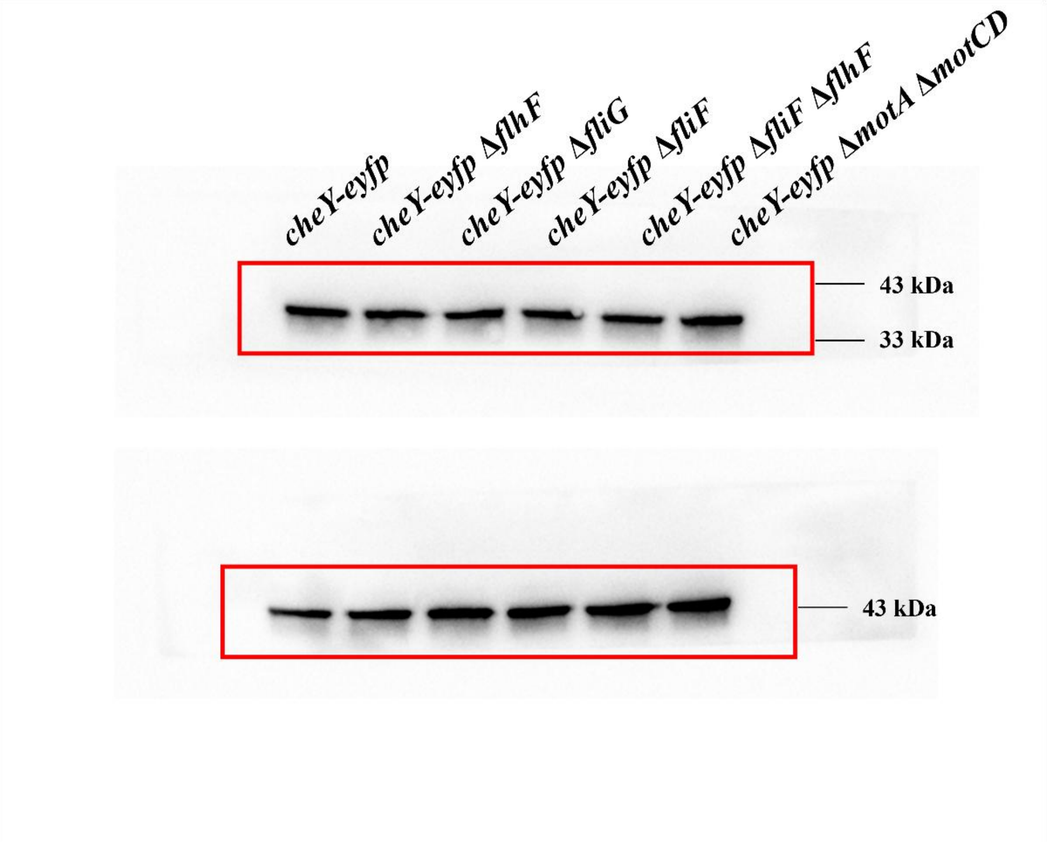

Figure 3B-source data 1. Original membranes (highlighted by red rectangular frames) corresponding to Figure 3B. The upper and lower bands represented the expression levels of protein CheY-eyfp and the housekeeping protein β-actin, respectively, with bands from left to right indicating the corresponding protein expression profiles of six bacterial strains wild-type, mutant Δ*flhF*, Δ*fliG*, Δ*fliF*, Δ*fliF* Δ*flhF*, and Δ*motA* Δ*motCD*.
