# Supplementary figures and images for: Spatial integration of sensory input and motor output in Pseudomonas aeruginosa chemotaxis through colocalized distribution
Source: eLife. 2025 Sep 4;13:RP97514. doi: 10.7554/eLife.97514 (PMC12410967; doi:10.7554/eLife.97514)

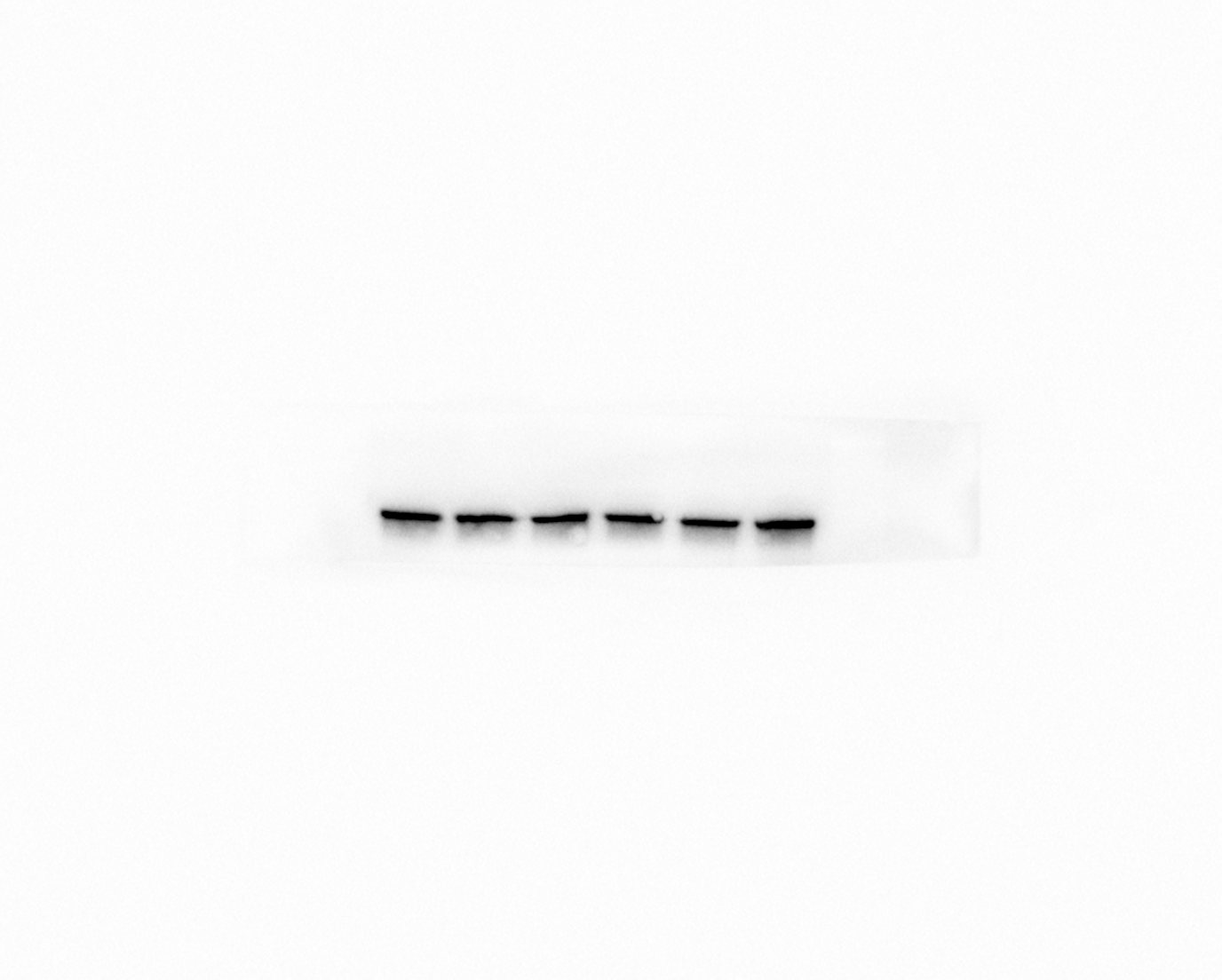

Supplement: Figure 3—source data 2. [file elife-97514-fig3-data2.zip › Figure 3B_Source data 2/CheY-eyfp.Tif]

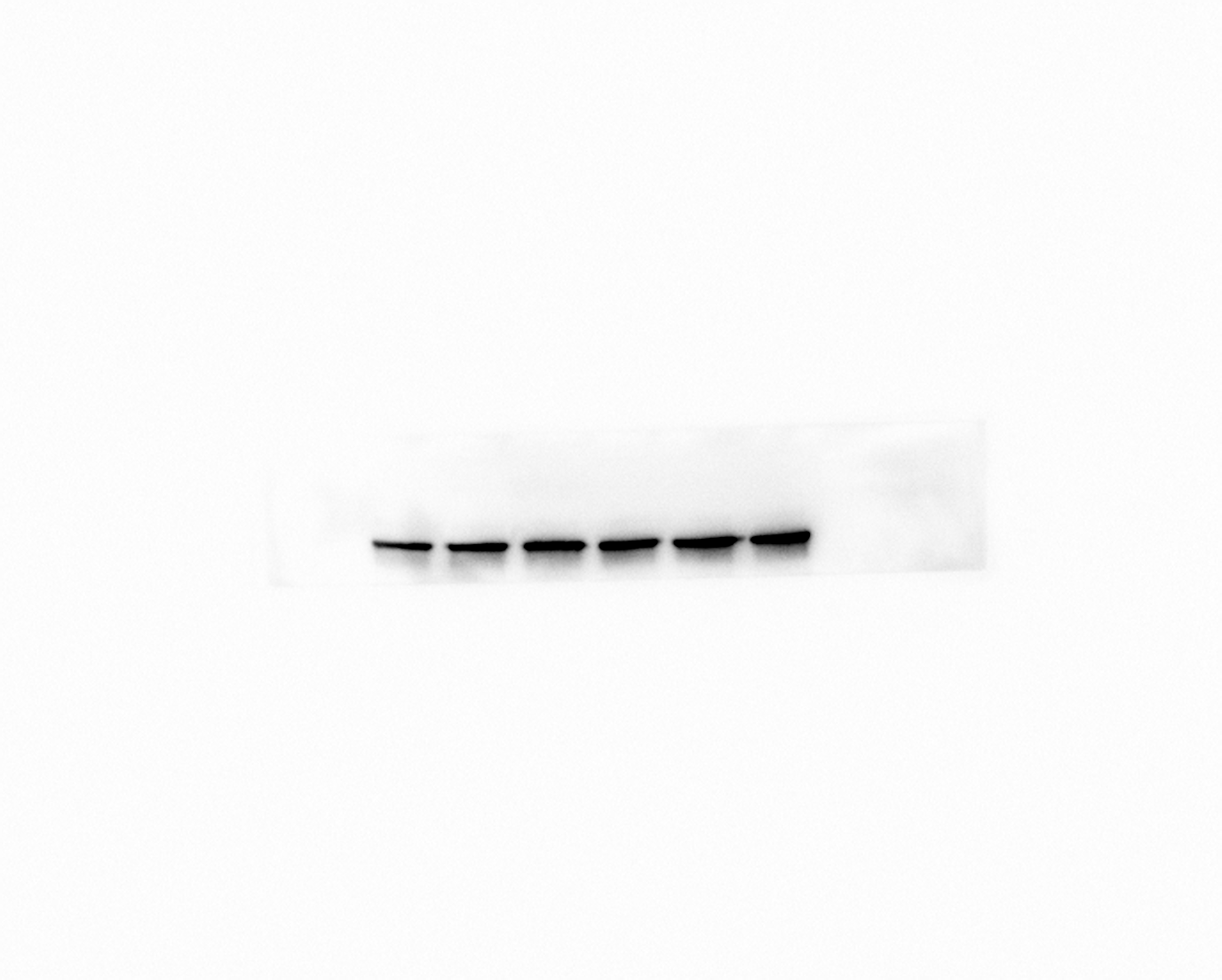

Supplement: Figure 3—source data 2. [file elife-97514-fig3-data2.zip › Figure 3B_Source data 2/β-actin.Tif]

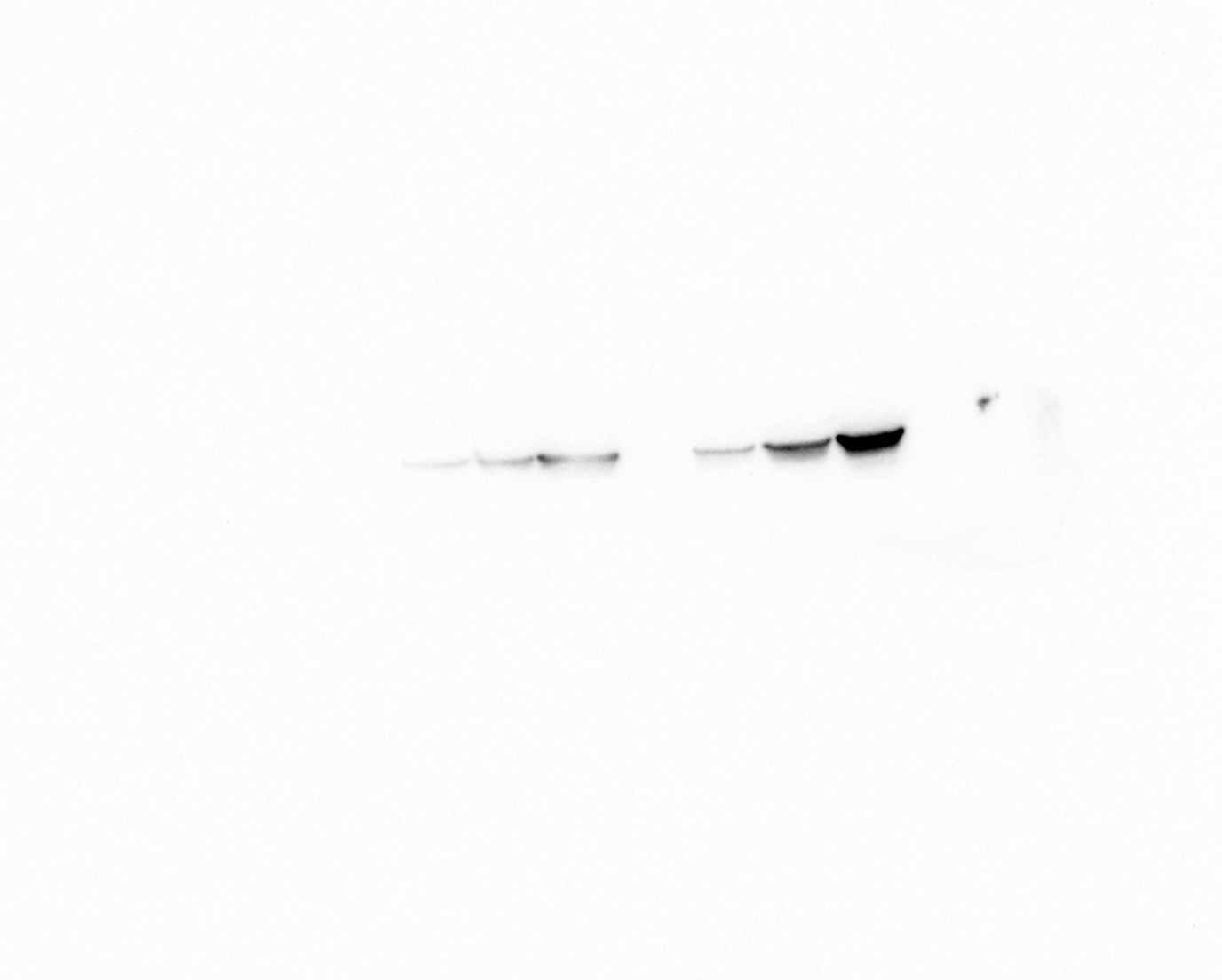

Supplement: Figure 4—figure supplement 1—source data 2. [file elife-97514-fig4-figsupp1-data2.zip › Figure 4-supplement 1-Source data 2/CheY-eyfp.Tif]

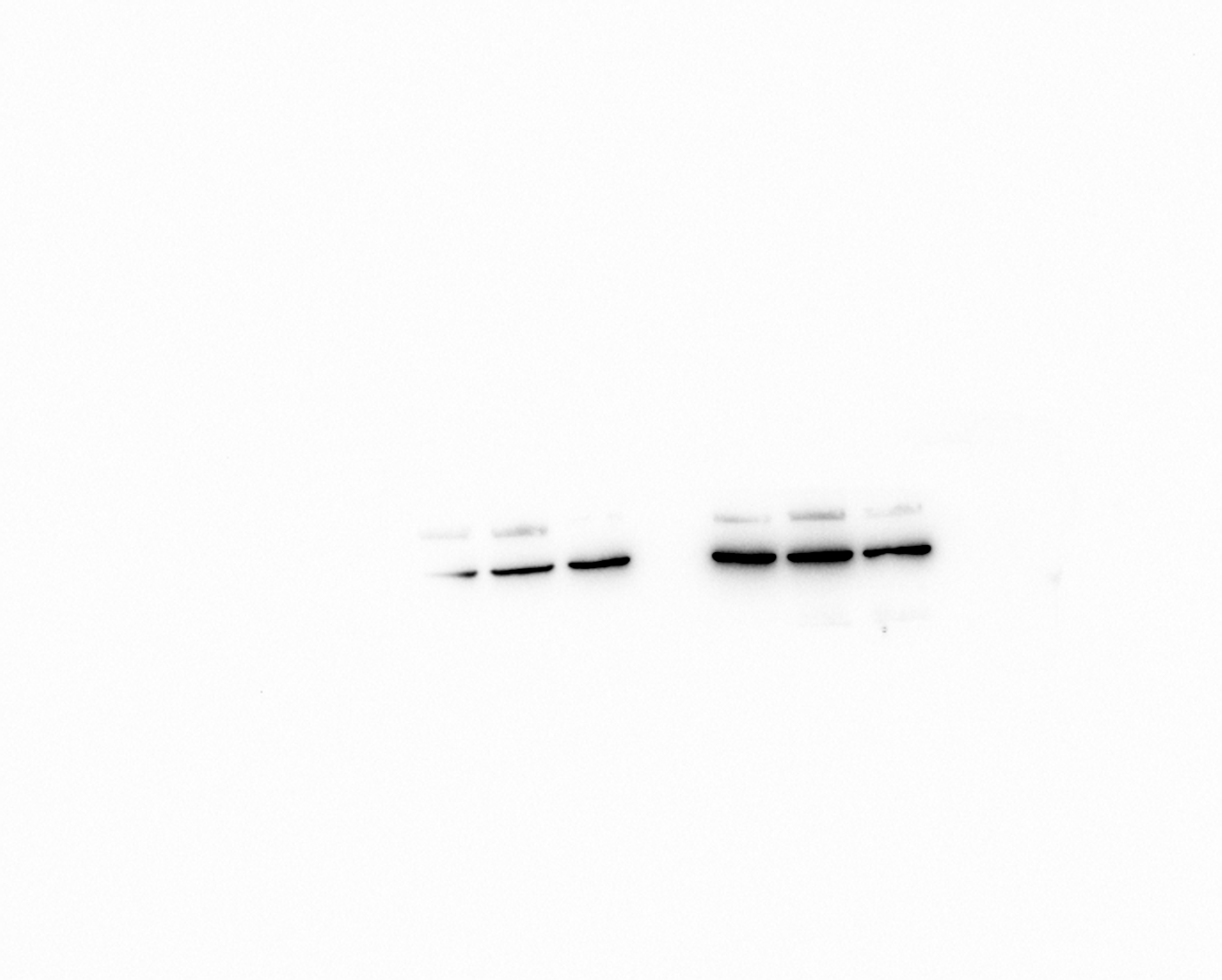

Supplement: Figure 4—figure supplement 1—source data 2. [file elife-97514-fig4-figsupp1-data2.zip › Figure 4-supplement 1-Source data 2/β-actin.Tif]
