# Supplementary material for: Spatial integration of sensory input and motor output in Pseudomonas aeruginosa chemotaxis through colocalized distribution
Source: eLife. 2025 Sep 4;13:RP97514. doi: 10.7554/eLife.97514 (PMC12410967; doi:10.7554/eLife.97514)
Supplement: Figure 4—figure supplement 1—source data 1. [file elife-97514-fig4-figsupp1-data1.zip › Figure 4-supplement 1-Source data 1/Figure 4-figure supplement 1-source data 1.pdf]

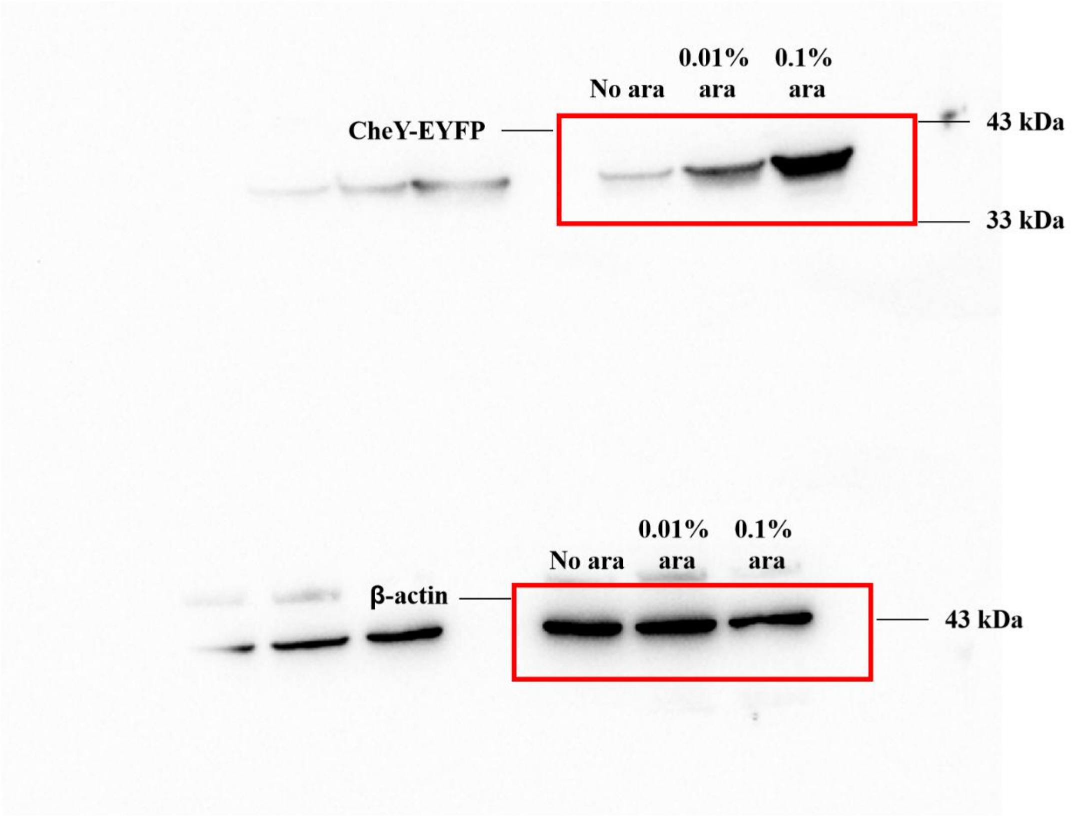

Figure 4-figure supplement 1-source data 1. Original membranes (highlighted by red rectangular frames) corresponding to Figure 4-figure supplement 1. The upper and lower bands represented the expression levels of protein CheY-eyfp and the housekeeping protein β-actin, respectively, with bands from left to right indicating the corresponding protein expression levels induced by different arabinose concentrations 0, 0.01, and 0.1 (%, m/v).
